# Deep learning-based diatom taxonomy on virtual slides

**DOI:** 10.1038/s41598-020-71165-w

**Published:** 2020-09-02

**Authors:** Michael Kloster, Daniel Langenkämper, Martin Zurowietz, Bánk Beszteri, Tim W. Nattkemper

**Affiliations:** 1 Department of Phycology, Faculty of Biology, University of Duisburg-Essen, Essen, Germany; 2 Biodata Mining Group, Faculty of Technology, Bielefeld University, Bielefeld, Germany

**Keywords:** Optical imaging, Marine biology, Software, Scientific data, Software, High-throughput screening

## Abstract

Deep convolutional neural networks are emerging as the state of the art method for supervised classification of images also in the context of taxonomic identification. Different morphologies and imaging technologies applied across organismal groups lead to highly specific image domains, which need customization of deep learning solutions. Here we provide an example using deep convolutional neural networks (CNNs) for taxonomic identification of the morphologically diverse microalgal group of diatoms. Using a combination of high-resolution slide scanning microscopy, web-based collaborative image annotation and diatom-tailored image analysis, we assembled a diatom image database from two Southern Ocean expeditions. We use these data to investigate the effect of CNN architecture, background masking, data set size and possible concept drift upon image classification performance. Surprisingly, VGG16, a relatively old network architecture, showed the best performance and generalizing ability on our images. Different from a previous study, we found that background masking slightly improved performance. In general, training only a classifier on top of convolutional layers pre-trained on extensive, but not domain-specific image data showed surprisingly high performance (F1 scores around 97%) with already relatively few (100–300) examples per class, indicating that domain adaptation to a novel taxonomic group can be feasible with a limited investment of effort.

## Introduction

Diatoms are microscopic algae possessing silicate shells called frustules^1^. They inhabit marine and freshwater environments as well as terrestrial habitats. Taxonomic composition of their assemblages is routinely assessed using light microscopy in ecological, bioindication and paleoclimate research^2–4^. Silicate frustules cleaned of organic material and embedded into high refractive index mountant on cover slips represent the most widely used type of microscopic preparations for such analyses^5,6^. Attempts to computerize parts or the whole of this workflow have been made repeatedly, starting with Cairns^7^, and in the most complete manner so far by the ADIAC project^8^, motivated by the desire to speed up the taxonomic enumeration process, to reduce its dependence on highly trained taxonomic experts, and to make identification results more reproducible and transparent. Recently, we described an updated re-implementation of most parts of this workflow, covering high throughput microscopic imaging, segmentation and outline shape feature extraction of diatom specimens^9,10^ which we since mainly applied in morphometric investigations^11–14^. The missing component in this workflow has been automated or computer-assisted taxonomic identification.

For this purpose, i.e. image classification in a taxonomic context, deep convolutional neural networks (CNNs) are currently becoming the state-of-the-art technique. Due to the broadening availability of high throughput, in part, in situ, imaging platforms^15–18^, and large publicly available image sets^19^, marine plankton has probably been addressed most commonly in such attempts in the aquatic realm^20–23^. The attention, however, recently also broadened to fossil foraminifera^24^, radiolarians^25^, as well as diatoms^26,27^. Due to the availability of deep learning software libraries^28,29^, well performing network architectures pre-trained on massive data sets like ImageNet^30^, and experiences accumulating related to transfer learning, i.e., application of pre-trained networks upon smaller data sets from a specialized image domain, the utilization of deep CNNs for a particular labelled image library is now within reach of taxonomic specialists of individual organismic groups.

In the case of diatom analyses, most studies thus far have addressed individual aspects of the taxonomic enumeration workflow in isolation, such as image acquisition by slide scanning^31^, diatom detection, segmentation and contour extraction^9,32,33^, or taxonomic identification^26,34,35^. Although all these aspects have been considered in detail by ADIAC^8^ and, with the exception of the final identification step, in our recent work^10^, a practicable end-to-end digital diatom analysis workflow has not emerged thus far. In this work, we introduce substantial further developments to these previously described workflows, now covering all aspects from imaging to deep learning-based classification, and apply it in a low diversity diatom habitat, the pelagic Southern Ocean, harbouring a unique and paleo-oceanographically and biogeochemically interesting diatom flora^36–40^.

The so far most extensive work on the application of deep learning on taxonomic diatom classification from brightfield micrographs^26^ tested only one CNN architecture to investigate the effects of training set size, histogram normalization and a coarse object segmentation that aimed more for a figure ground separation than for an exact segmentation.

We propose a procedure combining high resolution focus-enhanced light microscopic slide scanning, web-based taxonomic annotation of gigapixel-sized “virtual slides”, and highly customized and precise object segmentation, followed by CNN-based classification. In a transfer learning experiment employing a full factorial design varying CNN architecture, data set size, background masking and out-of-set testing (i.e. using data from different sampling campaigns for training and prediction), we address the questions (1) how well do different CNN architectures perform on the task of diatom classification; (2) to what extent does the increase in the size of training image sets improve transfer learning performance; (3) to what extent does a precise segmentation of diatom frustules influence classification performance; (4) to what extent is a CNN trained on one sample set (in this case, expedition) applicable to samples obtained from a different set.

## Material and methods

### Sampling and preparation

Samples were obtained by 20 µm mesh size plankton nets from ca. 15 to 0 m depth during two summer Polarstern expeditions ANT-XXVIII/2 (Dec. 2011–Jan. 2012, https://pangaea.de/?q=ANT-XXVIII%2F2) and PS103 (Dec. 2016–Jan. 2017, https://pangaea.de/?q=PS103). In both cases, a north to south transect from around the Subantarctic Front into the Eastern Weddell Sea was sampled, roughly following the Greenwich meridian, covering a range of Subantarctic and Antarctic surface water masses. To obtain clean siliceous diatom frustules, the samples were oxidized using hydrochloric acid and potassium permanganate after Simonsen^41^ and mounted on coverslips on standard microscopic slides in Naphrax resin (Morphisto GmbH, Frankfurt am Main, Germany).

### Digitalization

**Figure 1:**
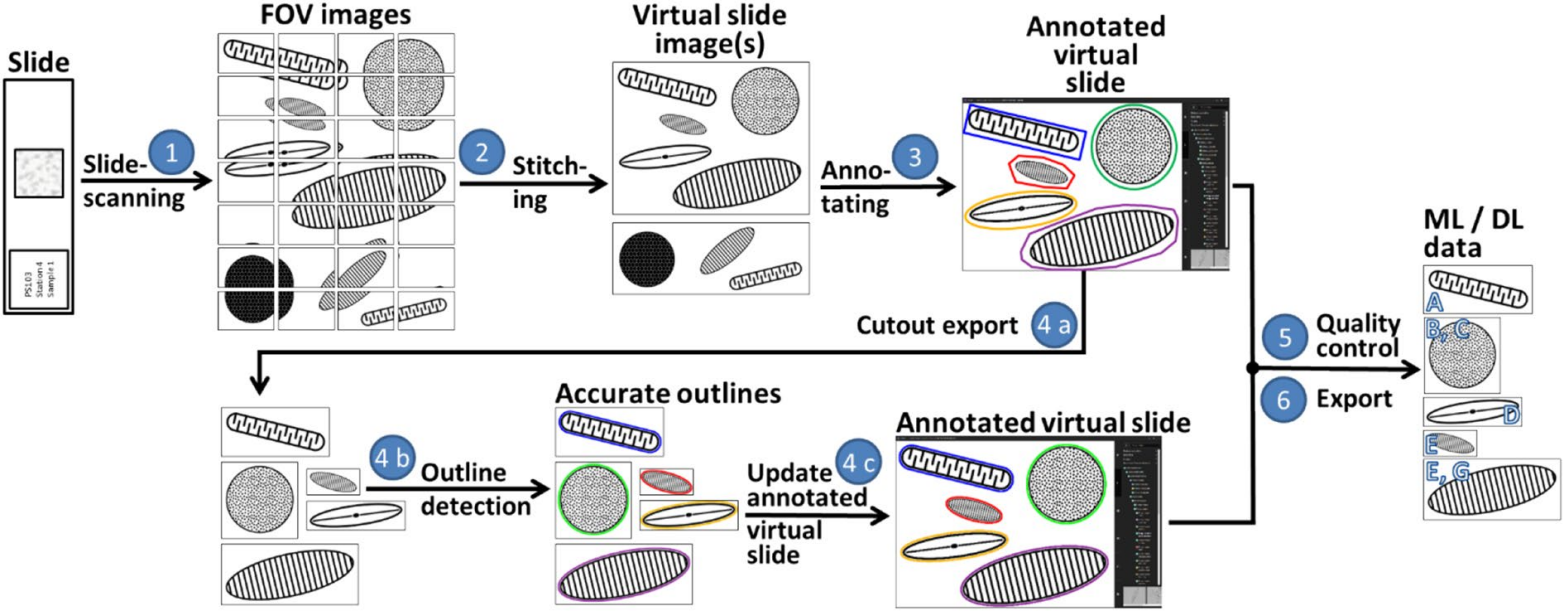
Workflow for generating annotated machine learning/deep learning data from physical slide specimens. Slides are scanned using a high resolution oil immersion objective as overlapping fields-of-view (1), those are stitched together to virtual slide images (2), which are uploaded to BIIGLE for annotating objects of interest (3), in our case diatom valves. The manually defined, rough object outlines can optionally be refined making use of SHERPA and SHERPA2BIIGLE (4a-c). After quality control using BIIGLE Largo or SHERPA2BIIGLE (5), cut-outs of annotated areas were exported along with label data (6) to assemble machine/deep learning data sets.

For converting these physical diatom samples into digital machine/deep learning data sets, we developed an integrated workflow consisting of the following steps (numbering refers to Fig. 1)

1. Slide scanning: Utilizing brightfield microscopy, we imaged a continuous rectangular region per slide, mostly ca. 5 × 5 mm^2^, in the form of several thousand overlapping field-of-view images (FOVs), where the scanned area usually contained hundreds to thousands of individual objects, mostly diatom frustules. For each FOV, at distances of one µm each, 80 focal planes were imaged and combined into one focus-enhanced image to overcome depth of field limitations. This technique is referred to as focus stacking and allows to observe a frustule's surface structure as well as its outline at the same time. Scanning and stacking were performed utilizing a Metafer slide scanning system (MetaSystems Hard & Software GmbH, Altlussheim, Germany) equipped with a CoolCube 1 m monochrome CCD camera (MetaSystems GmbH) and a high resolution/high magnification objective (Plan-APOCHROMAT 63x/1.4, Carl Zeiss AG, Oberkochen, Germany) with oil immersion (Immersol 518 F, Carl Zeiss AG, Oberkochen, Germany). This resulted in FOV images of 1,360 × 1,024 pixels at a resolution of 0.10 × 0.10 µm^2^/pixel. Device-dependent settings are detailed in^10^.
2. Slide stitching: The several thousand individual FOVs obtained during step 1 were combined into so-called virtual slides, gigapixel images capturing large portions of the scanned microscope slide at a resolution of ca. 0.1 × 0.1 µm^2^ per pixel. These were produced by a process called stitching, for which we used two different approaches. The Metafer VSlide Software (version 1.1.101) was applied to the PS103 scans, but produced misalignment artefacts (see Supplement I), frequently creating so-called ghosting (objects appeared doubled and shifted by a few pixels) and sometimes substantial displacement of FOVs, causing parts of the virtual slide image missing. As a consequence, during the course of the project we developed a method combining two ImageJ/FIJI plugins, MIST^42^ for exact alignment of FOV images and Grid/collection stitching^43^ for blending them into one large virtual slide image, which led to less stitching artefacts specifically for diatom slides. The scans from ANT-XXVIII/2 were processed using this stitching method.
3. Collaborative annotation: The virtual slide images were uploaded to the BIIGLE 2.0 web service^44^ for collaborative image annotation of objects of interest (OOIs). This term refers to all object categories used in the labelling process, in our case diatom frustules and valves (the frontal plates of a frustule) from various species and genera, diatom girdlebands (the radial parts of a frustule) and silicate skeletons or shells of non-diatom organisms like silicoflagellates or radiolarians. The OOIs were marked manually using BIIGLE’s annotation tool. Since following object boundaries precisely is very time-consuming, the OOI outlines were sketched only very roughly by a bounding box or a simple polygonal approximation. Objects distorted substantially by stitching artefacts (see step 2) were skipped. Predefined labels, mostly specifying names of Southern Ocean diatom taxa imported from WORMS^45^, were attached by four users, two of them (B.B., M.K.) being among the authors of this work. In some cases, multiple users annotated the same object, either agreeing or disagreeing on previously attached labels, where taxonomic disagreement is not uncommon^11^. This issue was resolved during data export (step 6).
4. Outline refinement: The roughly marked object outlines were refined to the exact object shape utilizing the semi-automatic segmentation feature of the diatom morphometry software SHERPA^9^. To this end, cut-outs depicting individual OOIs were produced from the virtual slide images by our software SHERPA2BIIGLE (step 4a). These cut-outs were processed with SHERPA for computing the actual object outlines, where faulty segmentations were refined manually (step 4b). Using another function of SHERPA2BIIGLE, these accurate segmentations were then uploaded into BIIGLE to replace the roughly marked object outlines (step 4c).
5. Quality control: The BIIGLE Largo^46^ feature (see Supplement II), as well as SHERPA2BIIGLE, were used to validate annotated labels. BIIGLE Largo enabled inspecting a large number of objects marked with a certain label simultaneously by displaying a series of scaled-down thumbnail images, whilst SHERPA enabled scrutinizing such objects one at a time at their original resolution/screen size. Erroneous label assignments were then corrected.
6. Data export: For each annotated OOI, a rectangular cut out was extracted with a minimum margin of 10 pixels, utilizing SHERPA2BIIGLE. Cut-outs were produced with and without background masking in order to study the effect on the classification (see below). If background masking was applied, the background (i.e. the area outside the annotated object) was replaced by the average background grey value, with a smooth transition close to the object boundary. Labels and metadata were exported in CSV format. Downstream processing was executed by R^47^ scripts (R version 3.6.1). The most important steps here were filtering annotations according to specific labels and defining the gold standard if multiple diverging labels had been attached to the same object, in which case the label attached by the senior expert (B.B.) was used.

### Data

**Figure 2:**
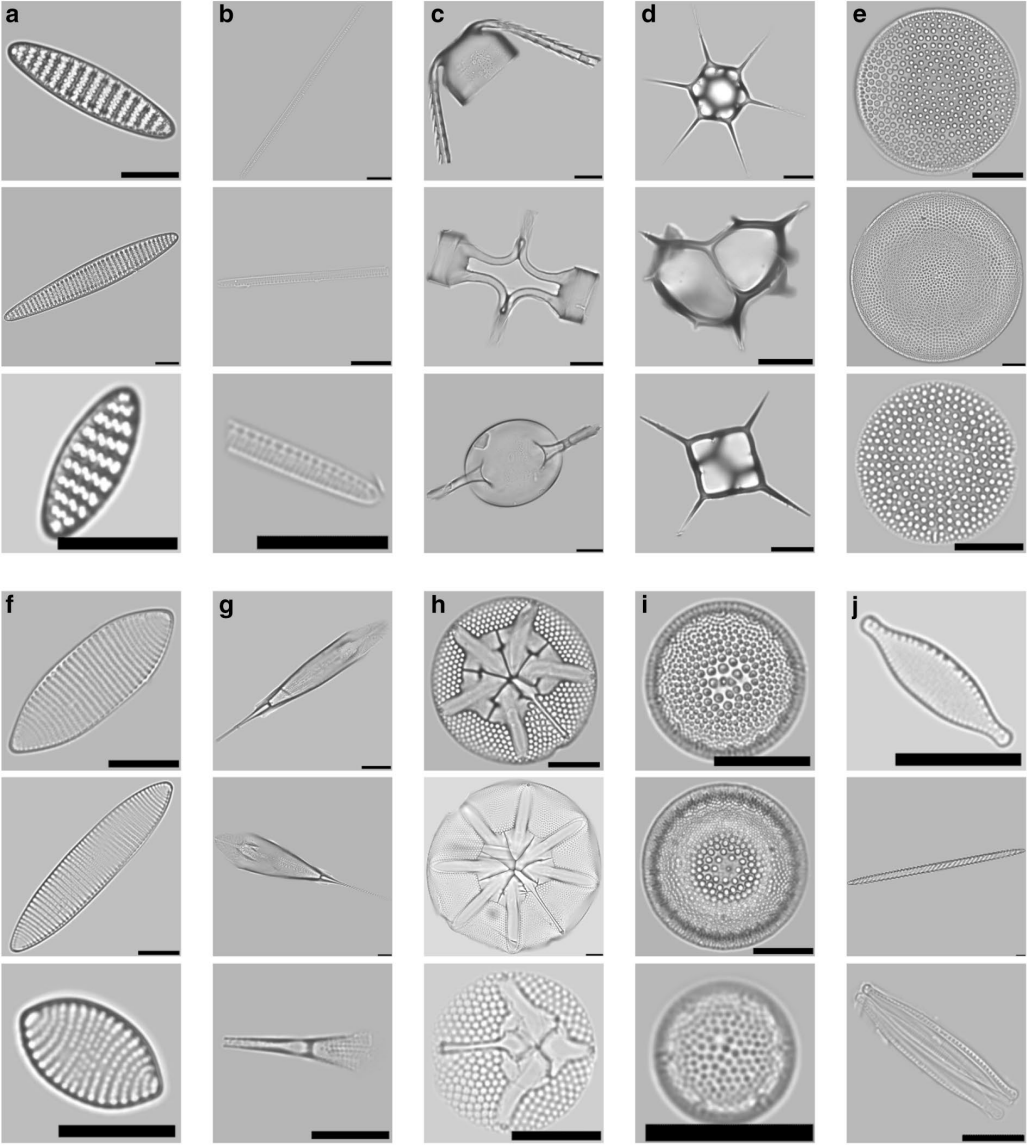
Three typical representatives of each annotated class, illustrating their variability in size and morphology: (**a**) *Fragilariopsis* *kerguelensis*, (**b**) *Pseudonitzschia*, (**c**) *Chaetoceros*, (**d**) Silicoflagellate, (**e**) *Thalassiosira* *lentiginosa*, (**f**) *Fragilariopsis* *rhombica*, (**g**) *Rhizosolenia*, (**h**) *Asteromphalus*, (**i**) *Thalassiosira* *gracilis*, (**j**) *Nitzschia*. Black scale bars represent a width of 100 pixels or 10.2 µm, resp.

**Figure 3:**
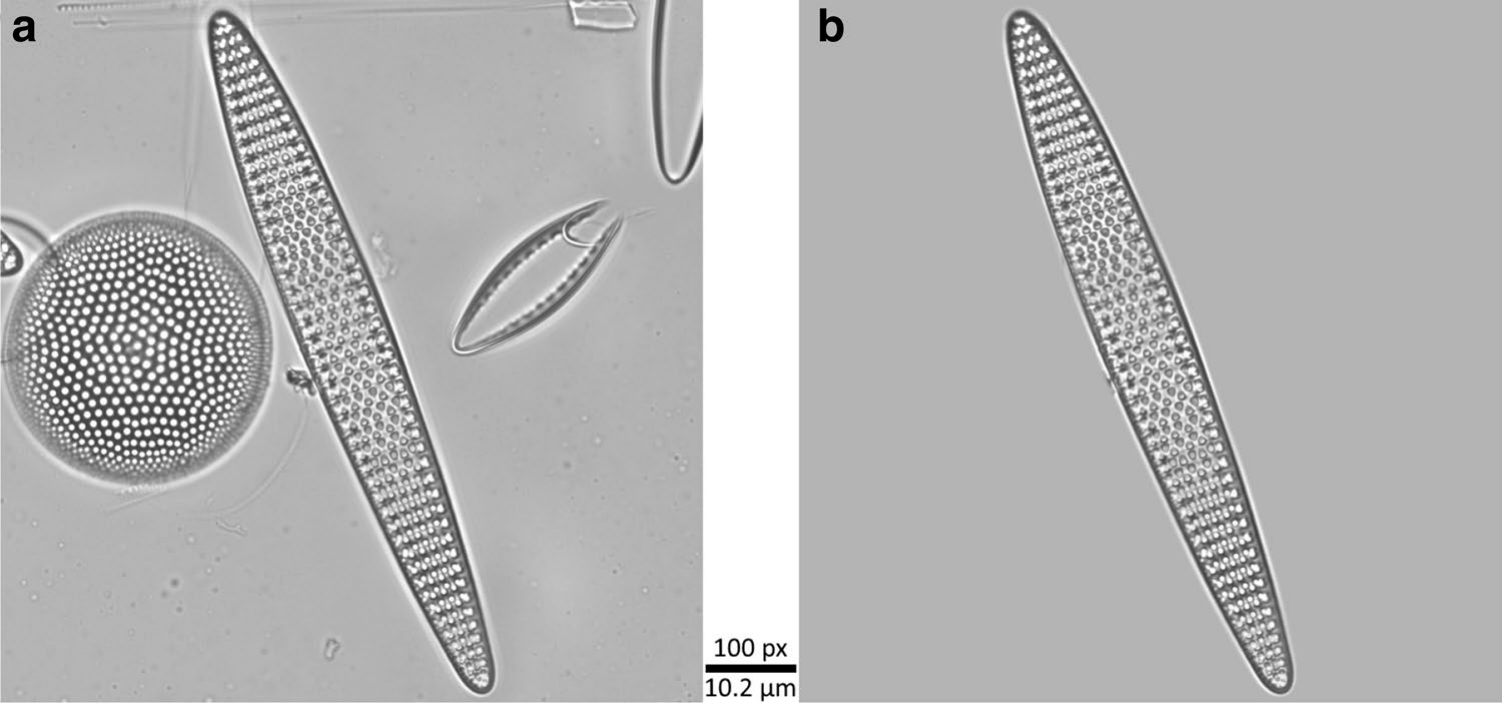
Example of a typical cut-out without (**a**) and with background masking (**b**). The diatom in the centre of both cut-outs is *Fragilariopsis* *kerguelensis.*

Using the protocol described above, we were able to collect annotations for nearly 10,000 OOIs of 51 different classes, originating from 26 virtual slides representing 19 physical slides. To allow for a sound comparison of the two expeditions ANT-XXVIII/2 and PS103, we limited this work to those 10 classes for which at least 40 specimens were present in each of the expeditions. Objects representing the diatom *Fragilariopsis* *kerguelensis*, which in the raw data accounted for nearly 40% of all specimens, were randomly subsampled to 660 specimens to reduce imbalance. This resulted in a total of 3,319 specimens from four diatom species, five diatom genera and the non-diatom taxon silicoflagellates (Table 1, Fig. 2). An example for a typical cut-out with and without background masking is given in Fig. 3.

**Table 1:** Base data set composition.

Class	n_total_	n_ANT-XXVIII/2_	n_PS103_
*Fragilariopsis* *kerguelensis*	660	418	242
*Pseudonitzschia*	520	173	347
*Chaetoceros*	434	82	352
Silicoflagellate	342	177	165
*Thalassiosira* *lentiginosa*	311	89	222
*Fragilariopsis* *rhombica*	272	57	215
*Rhizosolenia*	227	153	74
*Asteromphalus*	223	63	160
*Thalassiosira* *gracilis*	212	88	124
*Nitzschia*	118	76	42
∑	3,319	1,376	1,943

This enabled us to generate data sets (Table 2, Fig. 2) to design a deep learning-based classification. The data sets A_100,−_ (ANT-XXVIII/2, without background masking) and A_100,+_ (ANT-XXVIII/2, with background masking) contain 1,376 original cut-outs, the data sets B_100,−_ (PS103, without background masking) and B_100,+_ (PS103, with background masking) contain 1,943 original cut-outs (Table 1; instructions on downloading the original data are given in Supplement III). For splitting the data into training/validation/test sets, sampling was performed class-wise to ensure an even split of each class between the sets.

**Table 2:** Data set composition and denomination.

Expedition	ANT-XXVIII/2	PS103
Base data set (both expeditions merged), without background masking	AB_100,−_	
Full data set, without background masking	A_100,−_	B_100,−_
Full data set, with background masking	A_100,+_	B_100,+_
Data subset 10%, without background masking	A_10,−_	B_10,−_
Data subset 10% with background masking	A_10,+_	B_10,+_

### Experiments

The machine learning community continuously proposes new deep learning network architectures. In this work, we have tested a set of nine convolutional neural network architectures (Table 3)^48–53^. These were applied to learn diatom classification from the datasets described above using the KERAS default application models^54^. For each model, the pre-defined convolutional base with frozen weights pre-trained on ImageNet data^55^ was used as a basis and a new fully connected classifier module with a final softmax classification was trained on top of it. This approach usually is referred to as simple implementation of transfer learning without fine-tuning. Weights were adapted using the Adam optimizer^56^. We conducted the experiments utilizing the R interface to KERAS V2.2.4.1^57^. The input image intensity values were scaled to [0:1] and training data were augmented by rotation, shift, shear, zoom and flipping, using the functionality provided by the KERAS image data generator (see file “03-CNN functions.R” provided in Supplement III for details). The models were trained for 50 epochs, which for all investigated CNN architectures was sufficient to prohibit over- as well as underfitting. A batch size of 32 was used for the 100% data sets, and a batch size of 8 for the 10% data sets. All scripts were written in R and run on a Windows 10 system equipped with a nVidia Quadro P2000 GPU. R scripts are provided in Supplement III.

**Table 3:** CNN architectures.

Model	CNN convolutional base^a^	Classification layer(s) ^b^	Input shape
VGG16_1FC	VGG16	One 256 neuron dense layer	224 × 224
VGG16_2FC	VGG16	Two 256 neuron dense layers	224 × 224
VGG19_1FC	VGG19	One 256 neuron dense layer	224 × 224
VGG19_2FC	VGG19	Two 256 neuron dense layers	224 × 224
Xception	Xception	Global average pooling 2d	299 × 299
DenseNet	DenseNet	Global average pooling 2d	224 × 224
InceptionResNetv2	Inception-ResNet V2	Global average pooling 2d	299 × 299
MobileNetV2	MobileNet V2	Global average pooling 2d	224 × 224
InceptionV3	Inception V3	Global average pooling 2d	299 × 299

^a^Frozen, pre-trained on ImageNet data.

^b^Trained on our data for 50 epochs.

Classification performance was assessed by micro- and macro-averaged F1 scores according to

1 $${precision}_{class}= \frac{{TP}_{class}}{{TP}_{class}+{FP}_{class}}$$

2 $${recall}_{class}= \frac{{TP}_{class}}{{TP}_{class}+{FN}_{class}}$$

3 $${\mathrm{F}1}_{class}= \frac{2\times {precision}_{class}\times {recall}_{class}}{{precision}_{class}+{recall}_{class}}$$

4 $${precision}_{micro}= \frac{\sum TP}{\sum TP+\sum FP}$$

5 $${recall}_{micro}= \frac{\sum TP}{\sum TP+\sum FN}$$

6 $${\mathrm{F}1}_{micro}= \frac{2\times {precision}_{micro}\times {recall}_{micro}}{{precision}_{micro}+{recall}_{micro}}$$

7 $${\mathrm{F}1}_{macro}= \frac{\sum {\mathrm{F}1}_{class}}{{n}_{classes}}$$

With $TP$ = number of true positives, $FP$ = number of false positives, $FN$ = number of false negatives, ${n}_{classes}$ = number of classes. Subindex “$class$” refers to values per individual class, “$micro$” to micro-averaged values, “$macro$” to macro-averaged values.

If a class was not predicted (i.e. $TP$ = 0), ${precision}_{class}$ was set to 0 to allow for calculation of ${\mathrm{F}1}_{class}$.

**Figure 4:**
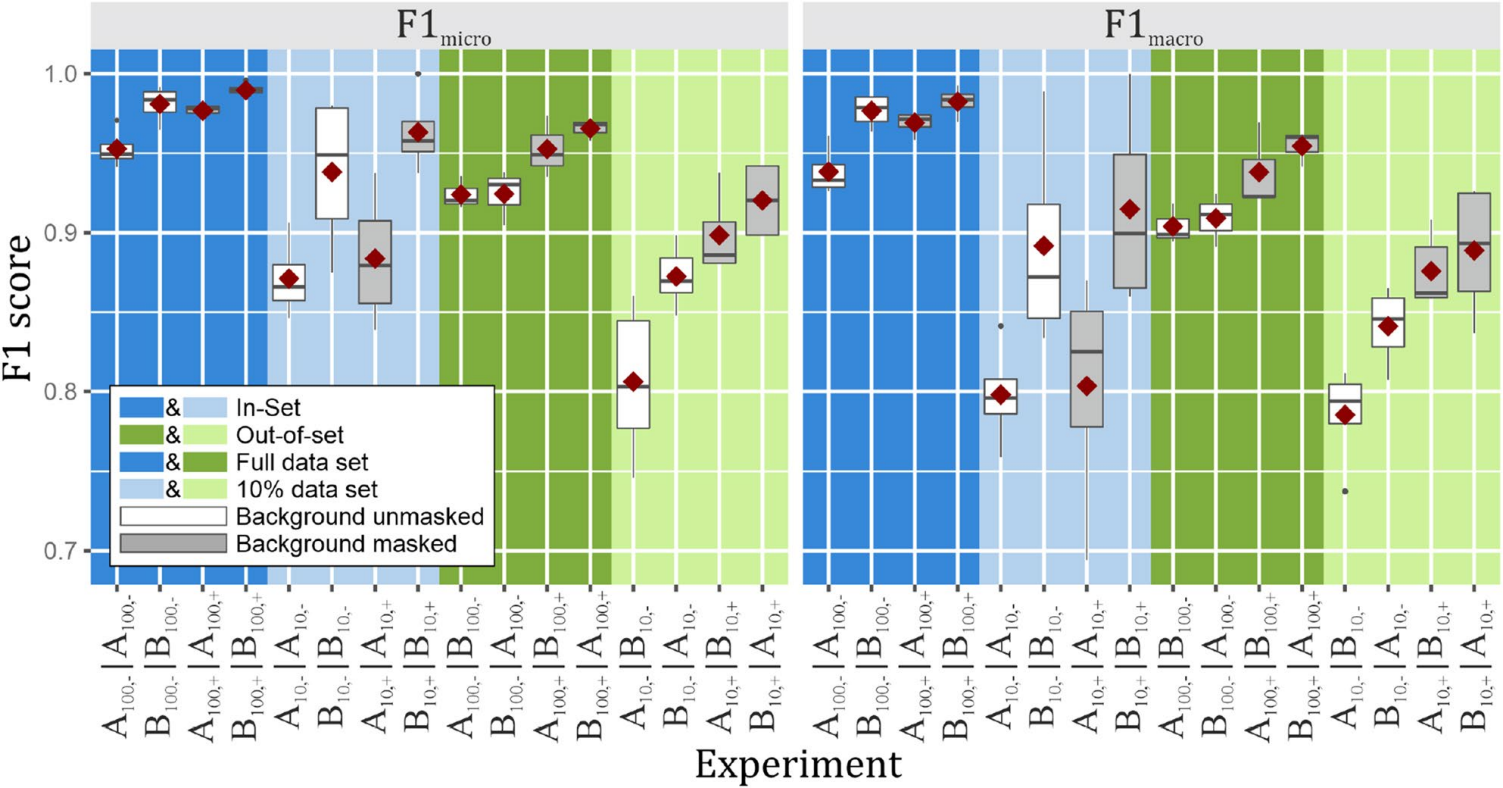
Boxplots comparing the classification performance for model “VGG16_1FC” experiments (Table 4). Mean values are indicated by red diamonds, black dots indicate outliers.

In order to address the questions raised in the introduction, we conducted 17 experiments (Table 4), one to test our data and setup and 16 to study the effects of (a) small/large number of training samples, (b) background masking and (c) possible concept drifts between data sets collected at similar geographic locations, but during different expeditions (i.e. in different years, Table 2, Fig. 4) and processed with different stitching methods. The individual data sets are referred to as shown in Table 2. The machine learning experiments and the results (as shown in Tables 4, 7, Fig. 4) are referred to as follows: the first part of the designation (left of “|”) denominates the data set used for training/validation, whilst the second part denotes the data set used for testing prediction performance. The test data were never used during the training and optimization of the network and thus are disjunct from the training/validation data, but were collected and prepared with identical parameters and conditions, with the exception of the expedition when indicated, which also implies application of a different stitching method.

**Table 4:** Deep learning experiments.

Row	Experiment	Training/validation data	Test data	Portion (%)	Background masking	Replication	Batch size
1	A_100,−_|A_100,−_	A	A	100	No	Fourfold cross validation	32
2	B_100,−_|B_100,−_	B	B	100	No		32
3	A_100,+_|A_100,+_	A	A	100	Yes		32
4	B_100,+_|B_100,+_	B	B	100	Yes		32
5	A_10,−_|A_10,−_	A	A	10	No		8
6	B_10,−_|B_10,−_	B	B	10	No		8
7	A_10,+_|A_10,+_	A	A	10	Yes		8
8	B_10,+_|B_10,+_	B	B	10	Yes		8
9	A_100,−_|B_100,−_	A	B	100	No	3 replicates	32
10	B_100,−_|A_100,−_	B	A	100	No		32
11	A_100,+_|B_100,+_	A	B	100	Yes		32
12	B_100,+_|A_100,+_	B	A	100	Yes		32
13	A_10,−_|B_10,−_	A	B	10	No	5 replicates	8
14	B_10,−_|A_10,−_	B	A	10	No		8
15	A_10,+_|B_10,+_	A	B	10	Yes		8
16	B_10,+_|A_10,+_	B	A	10	Yes		8

### Initial validation experiment

For testing our data and setup, the initial experiment “AB_100,−_|AB_100,−_” was conducted. This experiment represents the most commonly reported scenario where all of the data were used, i.e. all data from both expeditions were merged, and no background masking was applied. These data were split into 72% training, 18% validation and 10% test data, and a batch size of 32 was used for training the network.

### Experiments investigating influence of data set reduction, background masking and possible concept drift

Next, the two experiments “A_100,−_|A_100,−_” and “B_100,−_|B_100,−_” were conducted (Table 4 rows 1 and 2). To the data from each expedition without masking the image background (A_100,−_, B_100,−_), fourfold cross-validation was applied. For each of the four runs, the combined data from three folds (75% of the base data) were used to train the network. From these data, per class 80% were used for training and 20% for validating the training progress. The remaining fold (25% of the data) was used entirely to test classification performance.

Next, further intra-set experiments were conducted with modifications regarding background masking (Table 4 rows 3, 4, 7 and 8) and number of training data (rows 5–8). Data split and cross-validation were applied in the same way as in the first two experiments. The index “+” indicates that background masking was applied (cmp. Fig. 3), A_10_ (or B_10_) indicate that per class only 10% of the data were used for the entire experiment in order to simulate a significantly low number of training data. Accordingly, A_10,+_ refers to the experiment that uses only 10% of all data from ANT-XXVIII/2 to create training, validation and test set and where the background is masked in all the input images.

Subsequently, we investigated for the possible effect of transect-induced (expedition/year and stitching algorithm, respectively) concept drifts (Table 4 rows 9–16). Here, data from one expedition was used exclusively for training the network (split into 80% training and 20% validation data), and the trained network was applied to classify all cut-outs from data of the other expedition; we refer to this setup as “out-of-set” in the following. Experiments using the complete data sets (index “100”) were run with a batch size of 32 and three replications, for the reduced data sets (index “10”) a batch size of 8 was used with five replications Experiments.

### Downstream processing

The results were evaluated with R scripts, provided in Supplement III. Experiments were compared by analysis of variance (ANOVA), investigating the effects of CNN architecture, data set reduction, background masking and out-of-set prediction.

## Results

### Performance of different CNN architectures

From the variety of CNN models we investigated, those based on VGG architectures clearly outperformed the other models with respect to ${\mathrm{F}1}_{micro}$ and ${\mathrm{F}1}_{macro}$ values (Table 5, Eqs. (6) and (7)). In the following discussion, we will focus on the best performing model “VGG16_1FC” (VGG16 convolutional base with one downstream fully connected 256 neuron layer and a softmax classification layer). Detailed results of this model are provided in Supplement IV, a comprehensive comparison of all models is provided in Supplement V.

**Table 5:** Classification performance of different models, average over experiments 1–16, calculated by ANOVA.

Model	${\mathrm{F}1}_{micro}$	${\mathrm{F}1}_{macro}$
VGG16_1FC	0.92***	0.89***
VGG16_2FC	− 0.01	− 0.01
VGG19_1FC	− 0.01	− 0.01
VGG19_2FC	− 0.02	− 0.02
Xception	− 0.09***	− 0.12***
DenseNet	− 0.18***	− 0.19***
InceptionResNetV2	− 0.19***	− 0.26***
MobileNetV2	− 0.22***	− 0.31***
InceptionV3	− 0.23***	− 0.31***

F1 scores are calculated according to Eqs. (6) and (7). For VGG16_1FC absolute values are given (marked in bolt font), for other models values are relative to these. Significance codes: ***p < 0.001.

### General classification performance of model “VGG16_1FC”

For our initial experiment “AB_100,−_|AB_100,−_”, which utilized the merged complete data from both expeditions without background masking, ${\mathrm{F}1}_{micro}$ and ${\mathrm{F}1}_{macro}$ values of 0.97 were achieved. The classification performance was below average only for classes where specimens were represented solely on the genus level (Table 6, F1 values marked in bold), thus containing a very wide range of morphologies (cmp. Fig. 2 b, c, g, j).

**Table 6:** Classification performance per class for the initial experiment AB_100,−_|AB_100,−_.

Class	${TP}_{class}$	${FP}_{class}$	${FN}_{class}$	${precision}_{class}$	${recall}_{class}$	${\mathrm{F}1}_{class}$
*Asteromphalus*	24	0	0	1.00	1.00	1.00
*Chaetoceros*	43	4	2	0.91	0.96	**0.93**
*Fragilariopsis* *kerguelensis*	68	2	0	0.97	1.00	0.99
*Fragilariopsis* *rhombica*	28	0	1	1.00	0.97	0.98
*Nitzschia*	11	0	3	1.00	0.79	**0.88**
*Pseudonitzschia*	51	1	3	0.98	0.94	**0.96**
*Rhizosolenia*	24	2	0	0.92	1.00	**0.96**
Silicoflagellate	37	0	0	1.00	1.00	1.00
*Thalassiosira* *gracilis*	23	0	0	1.00	1.00	1.00
*Thalassiosira* *lentiginosa*	33	0	0	1.00	1.00	1.00

Calculations and naming according to Eqs. (1)–(3). F1 scores below average are marked in bold font.

### Influence of data set reduction

Intra-set experiments based on full data sets from the individual expeditions with background masking (A_100,+_|A_100,+_, B_100,+_|B_100,+_) in general achieved the best classification performance (Table 7 rows 3 and 4) and thus have been chosen as base-line for analyses of variance (ANOVA) investigating the effects of data set reduction, background masking and out-of-set prediction (Table 8). Here, reducing the data sets to 10% of the original size resulted in a substantial and significant decrease in classification performance (${\mathrm{F}1}_{micro}$ − 0.06, ${\mathrm{F}1}_{macro}$ − 0.12).

**Table 7:** Deep learning experiments, results for the best performing model “VGG16_1FC”.

Row	Experiment	${\mathrm{F}1}_{micro}$	${\mathrm{F}1}_{macro}$
1	A_100,−_|A_100,−_	0.95	0.94
2	B_100,−_|B_100,−_	0.98	0.98
3	A_100,+_|A_100,+_	0.98	0.97
4	B_100,+_|B_100,+_	**0.99**	**0.98**
5	A_10,−_|A_10,−_	0.87	0.80
6	B_10,−_|B_10,−_	0.94	0.89
7	A_10,+_|A_10,+_	0.88	0.80
8	B_10,+_|B_10,+_	0.96	0.91
9	A_100,−_|B_100,−_	0.92	0.90
10	B_100,−_|A_100,−_	0.92	0.91
11	A_100,+_|B_100,+_	0.95	0.94
12	B_100,+_|A_100,+_	0.97	0.95
13	A_10,−_|B_10,−_	**0.81**	**0.79**
14	B_10,−_|A_10,−_	0.87	0.84
15	A_10,+_|B_10,+_	0.90	0.88
16	B_10,+_|A_10,+_	0.92	0.89

Experiments: X|Y corresponds to data set X used for training/validation and Y for testing, with “A” = expedition ANT-XXVIII/2, “B” = expedition PS103 and indices “100” = full data set, “10” = 10% subset, “−“ = background unmasked, “ + ” = background masked (cmp. Table 4). F1 scores were calculated according to Eqs. (6) and (7) and averaged over the cross validation folds or the replicates, respectively. Highest/lowest F1 scores are marked in bold font.

**Table 8:** ANOVA results of F1 scores for model “VGG16_1FC” experiments (Table 4).

Factor interactions			${\mathrm{F}1}_{micro}$	${\mathrm{F}1}_{macro}$
Portion 10%	Background masked	Out-of-Set		
**✗**	**✓**	**✗**	0.98***	0.98***
**✗**	**✗**	**✗**	− 0.02	− 0.02
**✓**	**✓**	**✓**	0.01	0.05
**✓**	**✓**	**✗**	− 0.06**	− 0.12***
**✗**	**✓**	**✓**	− 0.02	− 0.03
**✓**	**✗**	**✓**	− 0.03	− 0.03
**✗**	**✗**	**✓**	− 0.02	− 0.02
**✓**	**✗**	**✗**	0.00	0.00

Base-line values for experiments without factor interactions (i.e. average of “A_100,+_|A_100,+_” and “B_100,+_|B_100,+_”) are highlighted in bold font, effects of factor interactions are given relative to this base-line. Significance codes: ***p < 0.001, **p < 0.01.

### Influence of background masking and possible concept drift

Interactions of other factors, i.e. background masking and out-of-set prediction, were obscured by the low sample sizes of the 10% data sets (Fig. 4 bright blue and green areas). As a consequence, some classes were represented with a very low number of examples in the test data. This resulted in a higher variance for the F1 scores. To overcome this impediment, we investigated the other factor interactions on experiments utilizing only the full data sets (Table 9). Here, out-of-set prediction as well as not executing background masking resulted in a significant decrease in classification performance (F1 scores ca. − 0.02).

**Table 9:** ANOVA results of F1 scores for model “VGG16_1FC” experiments utilizing only the full data sets (Table 4 rows 1–4 and 9–12).

Factor interactions		${F1}_{micro}$	${F1}_{macro}$
Background masked	Out-of-Set		
**✓**	**✗**	0.98***	0.98***
**✗**	**✗**	− 0.02*	− 0.02^**·**^
**✓**	**✓**	− 0.02**	− 0.03**
**✗**	**✓**	− 0.02	− 0.02

Base-line values for experiments without factor interactions (i.e. average of “A_100,+_|A_100,+_” and “B_100,+_|B_100,+_”) are highlighted in bold font, effects of factor interactions are given relative to this base-line. Significance codes: ***p < 0.001, **p < 0.01, *p < 0.05, · *p* < 0.1

## Discussion

This work applied newly developed methods for producing annotated image data for investigating the influence of a range of factors on deep learning-based taxonomic classification of light microscopic diatom images. These methods and factors are discussed in the following:

### Workflow

Our workflow covers the complete process of generating annotated image data from physical slide specimens in a user-friendly way. This is achieved by combining microscopic slide scanning, virtual slides, web-based (multi-)expert annotation and (semi-)automated image analysis. Scanning larger areas of microscopy slides instead of individual user-selected fields of view helps to avoid overlooking taxa at the object detection step and enables later re-analysis. Multi-user annotation, as implemented in BIIGLE, facilitates consensus-building and defining the gold standard in case of ambiguous labelling^11^.

### Data quality

Our workflow (Fig. 1) produced cut-outs of a high visual quality, with a resolution close to the optical limit and enhanced focal depth (Fig. 2). This allowed to investigate the very fine and intricate structures of diatom frustules, which usually are essential for taxonomic identification. The specimens contained a variety of problematic but typical cases, for example incomplete frustules (e.g. *Pseudonitzschia*); ambiguous imaging situations where multiple, sometime overlapping objects are included in the same cut-out (see Fig. 3a for an example); large intra-class variability of object size, which for larger specimens distorts local features by scaling them to different sizes when the cut-out is downsized to the CNN’s input size (for most classes); different imaging angles causing substantial changes in the specimens’ appearance (e.g. *Chaetoceros*, *Silicoflagellates* Fig. 2c,d); broad morphological variability within one class (e.g. *Asteromphalus*, *Silicoflagellates* Fig. 2d,h); and pooling of morphologically diverse species into the same class (e.g. genus *Nitzschia* Fig. 2j). The latter problem will of course be solved with the accumulation of more images covering different species of genus, but the rest will in a large part remain characteristic of diatom image sets. In the full data sets (A_100,−/+_, B_100,−/+_), individual classes were covered by ca. 40–400 specimens each. This represents an amount of imbalance that is not uncommon in taxonomic classification.

### Classification performance of different CNN architectures

For our experimental setup, which used transfer learning but re-trained only the classification layers, the relatively old VGG16 architecture^48^ clearly outperformed (Table 5) the newer CNNs Xception^50^, DenseNet^51^ Inception-ResNet V2^49^, MobileNet V2^52^ and Inception V3^53^. The reason for this interesting observation may be that the large number of model parameters in the VGG16 CNNs allows learning of models that differ in a large number of single not strongly correlated details. We assume that the reason for not observing overfitting, even though classifier modules of CNN architectures of different complexity were trained for the same number of epochs, might be owed our augmentation scheme. The models were trained exclusively on augmented versions of the original data, and since we used 7 randomly parameterized augmentation features (rotation, width shift, height shift, shear, zoom, horizontal flip and vertical flip) the input seems to be distinct enough for each epoch to prohibit overfitting during 50 epochs.

### Classification performance of VGG16_1FC

Using the most commonly reported scenario where all of the data were pooled (experiment AB_100,−_|AB_100,−_), the VGG16_1FC network achieved a classification success of 97% (F1 scores 0.97). This is slightly lower than the 99% accuracy reported by Pedraza et al.^26^ for their classification of 80 diatom classes. A possibly important difference between both data sets is the higher proportion of morphologically heterogeneous classes in our case. In terms of methods used, Pedraza et al. applied fine tuning of the feature extraction layers, a technique which was not tested in our experiments, because in our opinion a 97% classification success already is suitable for routine application, whilst re-training the convolutional base for further improvement would be very demanding in terms of computational costs. However, the CNN architectures used in both studies are different, so a direct comparison for drawing deeper conclusions at this point is difficult. It will be interesting to more systematically investigate the effects of these and other further factors on deep learning diatom classification in future studies. Classification performance of the other investigated VGG architectures, i.e. VGG16_2FC, VGG19_1FC and VGG19_2FC (Table 3), was slightly, but not significantly worse. Accordingly, we conclude that for only 10 classes, one 256 neuron fully connected layer is sufficient for processing the information from the convolutional base for the final softmax classification layer.

### Influence of data set size

It is a common observation in machine learning that larger training sets result in better classification performance (condition to a good labelling quality). Nevertheless, using a tenfold of data increased the classification performance by only 6% (${\mathrm{F}1}_{micro}$) to 12% absolute (${\mathrm{F}1}_{macro}$). From the factors we investigated, this is the most substantial improvement (Tables 8, 9), but it also comes at the highest costs. This once again underlines that the availability of training data usually is the most crucial prerequisite in deep learning. Nevertheless, in this study already sample sizes of mostly below 100 specimens per class resulted in 95% correct classifications (F1 scores ca. 0.95 for experiment A_100,−_|A_100,−_), an astonishingly good result underlining the value of using networks pre-trained on a different image domain in situations where the amount of annotated images is a bottleneck. Our observation is also in line with the results of Pedraza et al.^26^, indicating that slightly below 100 specimens (plus augmentation) per class might be taken as a desirable minimum number for future investigations.

### Influence of masking

Background masking improved the classification performance by ca. 2% absolute (Table 9), but required substantial efforts for exact outline computation. The improvement probably results from avoiding ambiguities in cases where multiple objects of different classes are contained within the same cut-out (Fig. 3a) or where the OOI’s structures have an only weak contrast compared to debris in the background (Fig. 2b). Contrasting our findings, in Pedraza et al.^26^ background-segmentation impaired classification performance slightly. We assume this might be due to their hard masking of the image background in black, which might introduce structures that could be misinterpreted as significant features by the CNN, whereas we tried to avoid adding artificial structures by blending the OOI’s surroundings softly into the homogenized background. A second difference possibly contributing to explaining this difference might be that nearby objects might be depicted in the cut-outs generated by our workflow. Such situations were presumably avoided in^26^ where the objects were cropped manually by a human expert. Looking at it this way, it could be said that the accurate soft masking we applied (Fig. 3) more than compensates for the difficulties caused by the less selective automated imaging workflow. An additional benefit of the exact object outlines produced by our workflow is their potential use for training deep networks performing instance segmentation like Mask R-CNN^58^, Unet^59^ or Panoptic-DeepLab^60^.

### Possible concept drift

We observed a decline in classification performance of ca. 2% absolute for out-of-set classification (Table 9). Though significant, this effect is minimal. This speaks for the efficiency of our standardization of sampling, imaging, processing (with the exception of the stitching method) and analysis. The still remaining small shift might represent either a slight residual methodological drift, or genuine biological signal, i.e. morphological variation due to changes in environmental conditions or sampled populations.

## Conclusion

We revisited the challenge of automation of the light microscopic analysis of diatoms and propose a full workflow including high-resolution multi-focus slide scanning, collaborative web-based virtual slide annotation, and deep convolutional network-based image classification. We demonstrated that the workflow is practicable end to end, and that accurate classifications (in the range of 95% accuracy/F1 score) are attainable already with relatively small training sets containing around 100 specimens per class using transfer learning. Although more images, as well as more systematic testing of different network architectures, still have a potential to improve on these results, this accuracy is already in a range that a routine application of the workflow for floristic, ecological or monitoring applications now seems within near reach.

## Supplementary information

Supplementary file1
