# Supplementary material for: Deep learning-based diatom taxonomy on virtual slides
Source: Sci Rep. 2020 Sep 2;10:14416. doi: 10.1038/s41598-020-71165-w (PMC7468105; doi:10.1038/s41598-020-71165-w)
Supplement: Supplementary file 1 — Supplementary file1 [file 41598_2020_71165_MOESM1_ESM.pdf]

Deep learning-based diatom taxonomy on virtual slides

Michael Kloster[1,2,*], Daniel Langenkämper[2], Martin Zurowietz[2], Bánk Beszteri[1], Tim W. Nattkemper[2]

[1]University of Duisburg-Essen, Faculty of Biology, Department of Phycology, Essen, Germany
[2]Bielefeld University, Faculty of Technology, Biodata Mining Group, Bielefeld, Germany
[*]michael.kloster@uni-due.de

Supplements

I. Stitching artefacts

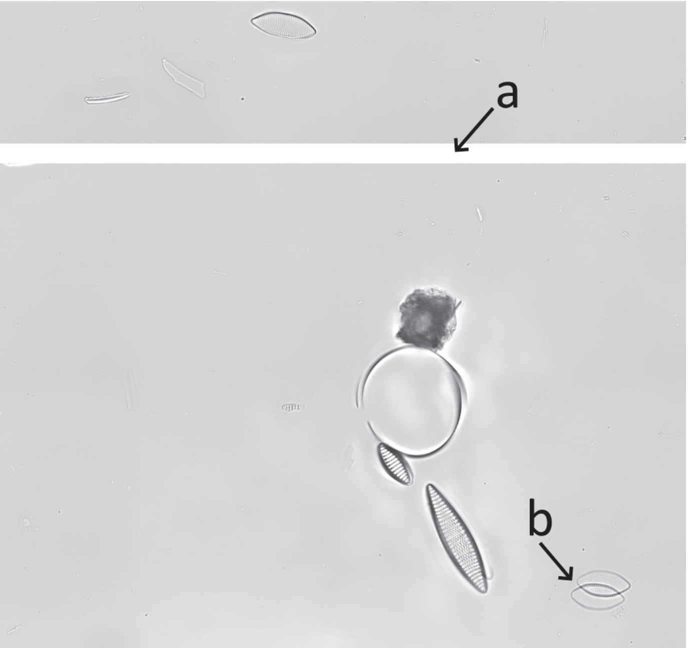

Figure S1: Typical stitching artefacts in virtual slides of PS103. a) missing scanning area, b) ghosting, i.e. doubling and shifting of objects

II. BIIGLE Largo

The BIIGLE Largo feature facilitates quick and effective quality control of annotations by inspecting thumbnail images of all objects marked with a certain label. This way, the general (morphological) concept of a class becomes clear and erroneous assignments can be identified visually.

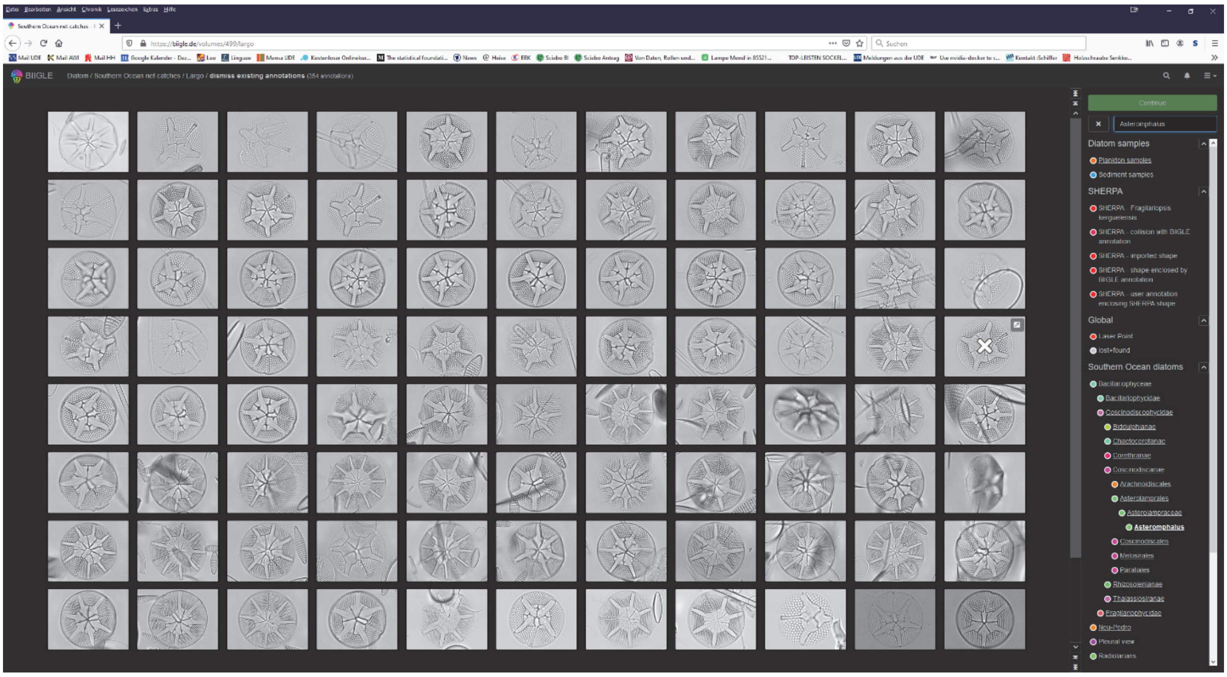

Figure S2: Screenshot of BIIGLE Largo. Thumbnails of objects marked with the same label are presented on the left side, the respective label is displayed on the right side.

III. Downloading data and R scripts

The data used for our experiments can be inspected at https://doi.pangaea.de/10.1594/PANGAEA.914544. A ZIP archive comprising all data (cut-out images as well as CSV files containing class labels, cut-out image filenames and metadata for the different experiments) can downloaded under the link “Annotated Southern Ocean diatom LM micrographs from PS79 & PS103 - download all” provided here.

A ZIP archive containing all data, our R scripts as well as F1 scores for all experiments be downloaded from https://www.uni-due.de/imperia/md/content/phykologie/supplement_iii_data__r_scripts_and_results.zip.

IV. Downloading detailed results for VGG16_1FC

Training histories and R console output of the experiments with the best performing CNN architecture VGG16_1FC can be downloaded from
https://www.uni-due.de/imperia/md/content/phykologie/supplement_iv_detailed_results_for_vgg16_1fc.zip.

V. Results for all investigated CNN architectures

Table S1: ANOVA of F1 scores for multiple models

	Data interactions				CNN Architecture							
	**Portion 10%**	**Background unmasked**	**Out-of-Set**	**VGG16_1FC**	**VGG16_2FC**	**VGG19_1FC**	**VGG19_2FC**	**Xception**	**DenseNet**	**InceptionResNetv2**	**MobileNetV2**	**InceptionV3**
$\mathbf{F1}_{micro}$	*All experiments*			*0.92****	*-0.01*	*-0.01*	*-0.02*	*-0.09****	*-0.18****	*-0.19****	*-0.22****	*-0.23****
	×	×	×	**0.98*****	**0.98*****	**0.98*****	**0.98*****	**0.94*****	**0.88*****	**0.81*****	**0.80*****	**0.82*****
	✓	×	×	-0.06**	-0.08***	-0.05**	-0.07***	-0.09***	-0.05	-0.07*	-0.07**	-0.07$^{\cdot}$
	×	✓	×	-0.02	-0.02	-0.02	-0.02	-0.10***	-0.06	-0.05	0.00	-0.01
	×	×	✓	-0.02	-0.03	-0.05**	-0.03	-0.04$^{\cdot}$	-0.17**	-0.05	-0.11***	-0.09*
	✓	✓	×	0.00	0.03	0.00	-0.03	0.01	0.00	0.01	-0.04	-0.06
	✓	×	✓	0.01	-0.01	-0.01	0.01	0.04	0.04	0.03	0.02	-0.03
	×	✓	✓	-0.02	0.00	0.02	-0.01	0.00	0.05	-0.03	0.00	0.00
	✓	✓	✓	-0.03	-0.04	-0.04	0.03	-0.03	-0.09	-0.02	0.00	-0.02
$\mathbf{F1}_{macro}$	*All experiments*			*0.89****	*-0.01*	*-0.01*	*-0.02*	*-0.12****	*-0.19****	*-0.26****	*-0.31****	*-0.31****
	×	×	×	**0.98*****	**0.98*****	**0.98*****	**0.97*****	**0.92*****	**0.83*****	**0.71*****	**0.68*****	**0.69*****
	✓	×	×	-0.12***	-0.13***	-0.09***	-0.12***	-0.15***	-0.12**	-0.11**	-0.13***	-0.13***
	×	✓	×	-0.02	-0.02	-0.02	-0.02	-0.15***	-0.05	-0.04	0.02	0.01
	×	×	✓	-0.03	-0.04	-0.07**	-0.04	-0.04	-0.13**	-0.02	-0.09**	-0.05
	✓	✓	×	0.00	0.03	-0.03	-0.06	0.01	0.02	0.02	-0.02	-0.05
	✓	×	✓	0.05	0.03	0.01	0.03	0.08*	0.11	0.08	0.03	-0.01
	×	✓	✓	-0.02	0.00	0.03	-0.01	0.01	0.05	-0.02	0.04	0.00
	✓	✓	✓	-0.03	-0.05	-0.01	0.04	-0.02	-0.13	-0.07	-0.03	-0.02

Rows "All experiments" give the average F1 score over all experiments as described in Fehler! Verweisquelle konnte nicht gefunden werden.**, the base level is defined by the VGG16_1FC model, for other CNN architectures the values are relative to this. Base levels of the F1 scores without interactions (i.e. averages from experiments "$A_{100,+}$|$A_{100,+}$ " and "$B_{100,+}$|$B_{100,+}$") are highlighted in bold font, results of the other experiments are given relative to these.**
